# Etiological and Pathological Investigation of Bacterial Pneumonia in Forest Musk Deer (*Moschus berezovskii*) Using a Novel Multiplex PCR Assay

**DOI:** 10.1155/vmi/8110272

**Published:** 2026-09-23

**Authors:** Cheng Chang, Xiaorui Zhang, Runlai Cao, Qilin Wang, Xiaoxu Wang

**Affiliations:** ^1^ Key Laboratory of Special Animal Epidemic Disease, Ministry of Agriculture and Rural Affairs, Jilin Provincial International Cooperation Key Laboratory for Science and Technology Innovation of Special Animal and Plants, Institute of Special Animal and Plant Sciences, Chinese Academy of Agricultural Sciences, Changchun 130112, China, agri.gov.cn

**Keywords:** *E. coli*, forest musk deer, *K. pneumoniae*, multiplex PCR, *P. aeruginosa*, *P. multocida*, pneumonia

## Abstract

Pneumonia is a major cause of mortality in forest musk deer (*M. berezovskii*) breeding, leading to significant economic losses. Conventional pathogen detection methods, such as bacterial isolation and culture, are time‐consuming and costly. This study aimed to develop a rapid, specific, and sensitive multiplex PCR assay for the simultaneous detection of four common bacterial pathogens. Specific primers were designed to target genes: *gyrB* for *Escherichia coli*, *PhoE* for *Klebsiella pneumoniae*, *LasI* for *Pseudomonas aeruginosa*, and *Kmt1* for *Pasteurella multocida*. The multiplex PCR system was optimized by adjusting the annealing temperature and primer concentrations, and its specificity, sensitivity, and reproducibility were evaluated. The technique successfully detected all four pathogens simultaneously, demonstrating high specificity and good reproducibility. The detection limits were 9.2 × 10^4^ CFU/mL for *E. coli*, 1.3 × 10^5^ CFU/mL for *K. pneumoniae*, 1.5 × 10^5^ CFU/mL for *P. aeruginosa*, and 3.7 × 10^4^ CFU/mL for *P. multocida*. Using this method, a total of 47 lung tissue samples from dead forest musk deer across four regions in China were analyzed, and results were compared with bacterial isolation and 16S rRNA sequencing. A high rate of mixed infections (74.5%) was observed, with *E. coli* showing the highest detection rate (80.8%), followed by *K. pneumoniae* (65.9%), while *P. aeruginosa* (40.4%) and *P. multocida* (40.4%) were less prevalent. Moreover, histopathological analysis confirmed typical pneumonia lesions. This method provides a reliable method for rapid detection of major bacterial pathogens in musk deer, supporting clinical diagnosis and epidemiological studies.

## 1. Introduction

The forest musk deer, a member of the family Moschidae, is primarily distributed across southwestern and central China [1, 2]. Musk secreted by adult males possesses high medicinal and commercial value, making the species economically important [3]. China produces more than 70% of the world’s musk supply [4], and driven by increasing market demand, the price of musk has risen dramatically from 60 RMB (about 7.5 USD) per gram in 2005 to 800 RMB (about 125 USD) per gram in 2020 [5]. Musk is often referred to as “soft gold,” and industries associated with forest musk deer—including musk production, hides, bones, and ecotourism—generate billions annually.

However, disease remains a major constraint on forest musk deer farming. Captive individuals are highly susceptible to respiratory tract infections, gastrointestinal disorders, and parasitic diseases [6]. Respiratory diseases, in particular, pose a significant threat, with an incidence reaching up to 39.4% [7]. Bacterial infections are recognized as the primary cause of pneumonia‐related mortality in forest musk deer, and pneumonia accounts for approximately 50% of all deaths in captive‐bred populations [8]. The major pathogens implicated include *E. coli* [9], *K. pneumoniae* [10], *P. aeruginosa* [11, 12], and *P. multocida* [13], all of which are zoonotic or potentially transmissible to other domestic animals [14–17]. Considering the high market value of forest musk deer—where the price of a breeding pair typically ranges from 50,000 to 80,000 RMB (about 7300 to 11,300 USD) [5]—pneumonia‐induced mortality causes substantial economic losses.

Accurate identification of bacterial pathogens is, therefore, essential for disease surveillance and clinical management. Although bacteriological isolation and culture remain the “gold standard,” they require 48–72 h and rely heavily on skilled personnel, making them impractical for rapid on‐farm diagnostics [18]. Advances in molecular diagnostics have highlighted multiplex PCR as a powerful tool capable of simultaneously amplifying multiple gene targets within a single reaction. This method provides several advantages, including high specificity, rapid turnaround time, low cost, and suitability for automation, in clinical and microbiological diagnostics [19]. Multiplex PCR has been widely applied for the rapid and simultaneous detection of bacterial pathogens in animals, including *E. coli*, *K. pneumoniae*, *P. aeruginosa*, and *P. multocida*, demonstrating its high sensitivity, specificity, and diagnostic value [20–23].

Despite these developments, no multiplex PCR assay has been established to detect the major bacterial pathogens associated with pneumonia in forest musk deer in a single PCR reaction. This gap hinders timely diagnosis and effective disease control in deer farming. Therefore, this study aimed to develop a reliable molecular diagnostic assay for the simultaneous detection of four bacterial pathogens associated with pneumonia in forest musk deer.

## 2. Materials and Methods

### 2.1. Clinical Isolates Used to Establish the Assay

Between September 2023 and March 2024, forest musk deer exhibiting severe respiratory symptoms that subsequently died were subjected to aseptic necropsy. A total of 22 lung tissue samples or respiratory swabs (obtained by swabbing the freshly cut surface of the lung tissue after aseptic necropsy) were collected from Linzhi City (Xizang Autonomous Region), Haibei Zang Autonomous Prefecture (Qinghai Province), Zizhou County (Shaanxi Province), and Miyaluo Town (Sichuan Province).

All sample processing procedures were conducted in a biological safety cabinet. Tissue specimens were aseptically sectioned, and the freshly exposed surfaces were streaked onto Brain Heart Infusion Agar (BHIA, using BHI powder from Hope Bio‐technology Co., Ltd, China, and agar powder from Solarbio Science & Technology Co., Ltd., China) solid medium, whereas swab specimens were directly streaked onto BHIA plates. The inoculated plates were incubated in an inverted position at 37°C for 24 h. Representative single colonies were subsequently selected and inoculated into BHIB, followed by incubation at 37°C with shaking at 180 rpm for 24 h. Following bacterial culture and isolation, the isolated colonies were identified by 16S rRNA sequencing [24, 25].

### 2.2. Primers Design

The target genes selected in this study exhibited both intraspecies conservation and interspecies specificity, thereby ensuring the accuracy of detection. Among them, a housekeeping gene was included, while the feasibility of the remaining genes as detection targets has also been validated in previous studies. To be specific, based on the gene sequence information available in GenBank for the *E. coli gyrB* gene [26], the *K. pneumoniae PhoE* gene [27], the *P. aeruginosa LasI* gene [28], and the highly conserved *P. multocida Kmt1* gene [29], primers targeting these specific genes were designed using Primer Premier Version 5.0 (PREMIER Biosoft International, Palo Alto, CA, USA). The primer sequences and specific reference IDs are shown in Table 1. All primers were synthesized by Sangon Biotech (Shanghai) Co., Ltd.

**TABLE 1:** Primers used in multiplex PCR and bacteria identification.

Bacteria	Target gene	Primer sequence (5’⟶3′)	Product size	Gene bank ID
*E. coli*	*gyrB*	F:TATGAAGGCGGCATCAAGGC	787 bp	KJ868400.1, KJ868399.1, KJ868398.1
		R:GACCGATACCACAGCCAAGC		
*K. pneumoniae*	*PhoE*	F:ACCTACCGCAACACCGACTTCTTC	558 bp	AF064793.1, AY062124.1, AF009172.1
		R:GTTGATGCCGAGTTTGTTATCGCTTT		
*P. aeruginosa*	*LasI*	F:ACAAGTTGCGTGCTCAAGTGTT	361 bp	KR020726.1, KR020725.1, KR020724.1
		R:AGCGTCTGGATGTCGTTCTGC		
*P. multocida*	*Kmt1*	F:TGAGTGGGCTTGTCGGTAGTCT	244 bp	OR248566.1, OR248565.1, OR248564.1
		R:CGCTCTGCCGTTAATGGCTTCA		
Bacteria	16S rRNA	F:AGAGTTTGATCMTGGCTCAG	1492 bp	
		R:GGTTACCTTGTTACGACTT		

### 2.3. Culture of Reference Strains and Determination of Bacterial Concentration

In the biosafety cabinet, 100 μL of each standard bacterial suspension—*E. coli* (ATCC25922), *K. pneumoniae* (ATCC700603), *P. aeruginosa* (ATCC27853), and *P. multocida* (ATCC12453)—was spread evenly onto BHIA agar solid medium and incubated at 37°C for 24 h. After incubation, a single colony of each bacterium was inoculated into heart infusion broth (BHIB) liquid medium and cultured at 37°C with shaking at 180 rpm for 24 h. After removal from the shaker, the cultures were immediately placed in an ice‐water bath for 30 min. The resulting cultures were subjected to 10‐fold serial dilutions, and the original bacterial concentrations were determined using the colony‐counting method.

### 2.4. Establishment of Multiplex PCR Reaction Conditions

The multiplex PCR system volume is 25 μL, consisting of 12.5 μL 2 × ES *Taq* MasterMix (Beijing Kangwei Century Biotechnology Co., Ltd., Beijing, China, Lot No. CW0690H), 4.5 μL of ddH_2_O, 0.5 μL of each primer stock solution (0.625 μM), and 1 μL of each bacterial culture template (4 μL in total).

The PCR reactions were carried out by a gradient PCR instrument (Analytik Jena (Beijing) Co., Ltd.). After optimization of the reaction conditions, the final multiplex PCR amplification protocol was established as follows: an initial denaturation at 94°C for 5 min, followed by 30 cycles of denaturation at 94°C for 30 s, annealing at 60°C for 30 s, and extension at 72°C for 30 s, with a final extension at 72°C for 5 min. Among the *Taq* Mastermix containing Es *Taq* DNA Polymerase (amplification efficiency: 2 kb/min), MgCl_2_ (3 mM/L), dNTP (400 μM/L), PCR stabilizer, and PCR enhancer.

### 2.5. Sensitivity Test of Multiplex PCR

100 μL of each of the four target bacterial cultures was mixed. Then, 100 μL of the mixed suspension was added to 900 μL of BHIB liquid medium and subjected to tenfold serial dilutions. This procedure was repeated, obtaining 9 mixed samples in all, and the resulting dilutions were used for subsequent multiplex PCR reactions.

### 2.6. Specificity Test of Multiplex PCR

Bacterial cultures of 13 species, *E. coli*, *K. pneumoniae*, *P. aeruginosa*, *P. multocida*, *Streptococcus pneumoniae*, *Proteus mirabilis*, *Enterococcus faecalis*, *Enterococcus thailandicus*, *Acinetobacter lwoffii*, *Acinetobacter baumannii*, *Bacillus polymyxa*, *Bacillus amyloliquefaciens*, and *Citrobacter freundii,* were used as templates for PCR amplification with the designed specific primers.

### 2.7. Multiplex PCR Preliminary Validation Analysis for Field Samples

From March 2024 to September 2025, dead forest musk deer from farms in Linzhi City, Haibei Zang Autonomous Prefecture, Zizhou County, and Miyaluo Town were identified during routine farm inspections conducted three times daily and immediately subjected to aseptic necropsy. Gross pathological changes were examined, and fresh lung tissues with obvious respiratory lesions were collected and transported to our laboratory under cold‐chain conditions. In total, 47 lung tissue samples were obtained. Each lung tissue sample was cut into small pieces (about 1 cm^3^) and added to 1 mL of BHIB liquid medium, followed by tissue homogenization. The supernatants were then subjected to multiplex PCR analysis.

To further verify the reliability of the multiplex PCR and exclude potential false positives, the submitted samples were also carried out bacteriological isolation and culture, as well as 16S rRNA sequencing.

### 2.8. Histopathological Analysis for Field Samples

Lung tissue samples collected from a 1.5‐year‐old female deer were subjected to hematoxylin and eosin (H&E) staining and examined for histopathological changes. Tissue section preparation was performed according to the method described by Slaoui et al. [30].

## 3. Results

### 3.1. Identification of the Prevalence of Bacterial Species in Clinical Samples

A total of 13 bacterial species were identified from the collected samples, among which *E*. *coli*, *K*. *pneumoniae*, *P*. *aeruginosa*, and *P*. *multocida* were the most prevalent pathogenic bacteria and were, therefore, selected as target pathogens for subsequent multiplex PCR assay development. Nine nontarget strains, including *S*. *pneumoniae*, *P*. *mirabilis*, *E*. *faecalis*, *E*. *thailandicus*, *A*. *lwoffii*, *A*. *baumannii*, *B*. *polymyxa*, *B*. *amyloliquefaciens*, and *C*. *freundii*, were also isolated from the diseased samples. All strains were subsequently purified, cultured, and preserved in the Animal Infectious Disease Prevention and Control Team.

### 3.2. Reference Bacterial Culture Concentration

The colony‐counting results showed that the standard bacterial concentrations were 2.3 × 10^9^ CFU/mL for *E. coli*, 3.2 × 10^9^ CFU/mL for *K. pneumoniae*, 3.8 × 10^9^ CFU/mL for *P. aeruginosa*, and 9.2 × 10^8^ CFU/mL for *P. multocida*.

### 3.3. Establishment of Multiplex PCR Conditions

**FIGURE 1:**
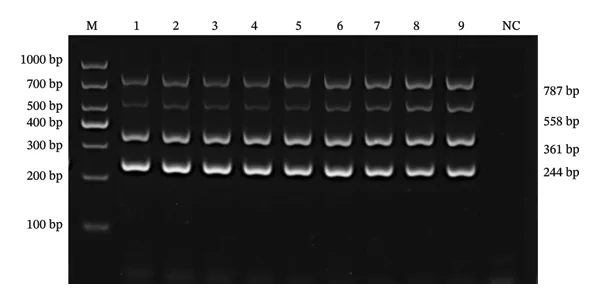
Results of multiplex PCR reaction at different annealing temperatures. Lane M: 1000 bp DNA marker; Lane 1: 55°C; Lane 2: 56°C; Lane 3: 57°C; Lane 4: 58.2°C; Lane 5: 60.6°C; Lane 6: 61.8°C; Lane 7: 63°C; Lane 8: 64°C; Lane 9: 65°C; NC: negative control.

**FIGURE 2:**
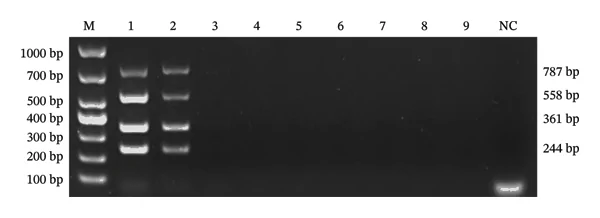
Results of multiplex PCR reaction using different primers concentrations. Lane M: 1000 bp DNA marker; Lane 1: 1.25 μM; Lane 2: 0.625 μM; Lane 3: 0.3125 μM; Lane 4: 156.25 nM; Lane 5: 78.125 nM; Lane 6: 39.06 nM; Lane 7: 19.53 nM; Lane 8: 9.77 nM; Lane 9: 4.88 nM; NC: negative control.

Each bacterium template showed that the specific gene amplification products were 787 bp for *E. coli*, 558 bp for *K. pneumoniae*, 361 bp for *P. aeruginosa*, and 244 bp for *P. multocida*. The annealing temperature had no significant effect on amplification, and the band clarity remained comparable across the 55°C–65°C range as shown in Figure 1. Therefore, an annealing temperature of 60°C was selected for subsequent experiments. In the multiplex PCR system, distinct amplification bands were obtained when the primer stock concentrations were 1.25 μM and 0.625 μM, whereas no bands were observed at lower primer concentrations, as demonstrated in Figure 2. Thus, the primer stock concentration of 0.625 μM was selected for subsequent experiments.

### 3.4. Results of Multiplex PCR Sensitivity Experiment

**FIGURE 3:**
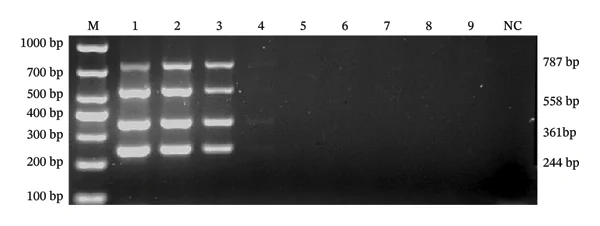
Results of multiplex PCR sensitivity experiment. Lane M: 1000 bp DNA marker; Lane 1–9: tested bacterium at different concentrations. NC: negative control.

The PCR results of the sensitivity experiment are shown in Figure 3 that *E. coli*, *K. pneumoniae*, *P. aeruginosa*, and *P. multocida* produced the faintest bands in Lane 4. Thus, the limits of detection in a 25 μL PCR reaction volume were 9.2 × 10^4^ CFU/mL for *E. coli*, 1.3 × 10^5^ CFU/mL for *K. pneumoniae*, 1.5 × 10^5^ CFU/mL for *P. aeruginosa*, and 3.7 × 10^4^ CFU/mL for *P. multocida*.

### 3.5. Results of Multiplex PCR Specificity Experiment

**FIGURE 4:**
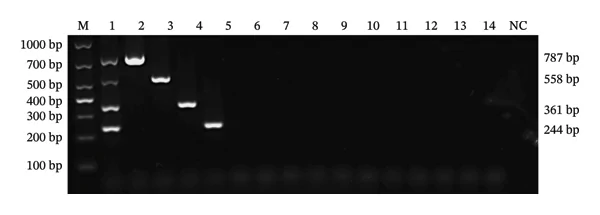
Results of multiplex PCR specificity experiment. Lane M: 1000 bp DNA marker; Lane 1: mixed sample of *E. coli*, *K. pneumoniae*, *P. aeruginosa*, and *P. multocida*; Lane 2: *E. coli*; Lane 3: *K. pneumoniae*; Lane 4: *P. aeruginosa*; Lane 5: *P. multocida*; Lane 6: *S. pneumoniae*; Lane 7: *P. mirabilis*; Lane 8: *E. faecium*; Lane 9: *E. thailandicus*; Lane 10: *A. lwoffii*; Lane 11: *A. baumannii*; Lane 12: *P. polymyxa*; Lane 13: *B. amyloliquefaciens*; Lane 14: *E. cloacae*. NC: negative control.

Figure 4 shows that *E. coli*, *K. pneumoniae*, *P. aeruginosa*, *and P. multocida* successfully produced the expected amplification products. No amplification was observed for *S. pneumoniae*, *P. mirabilis*, *E. faecium*, *E. thailandicus*, *A. lwoffii*, *A. baumannii*, *P. polymyxa*, *B. amyloliquefaciens*, or *E. cloacae*. These results indicate high primer specificity.

### 3.6. Results of Detecting and Identifying Analysis for Field Samples

**FIGURE 5:**
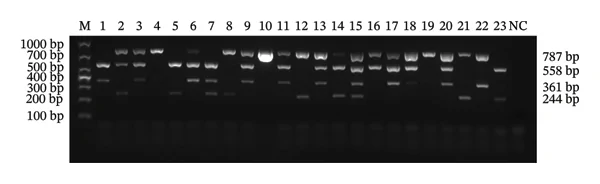
Results of detection and identification of randomly selected field samples. Lane M: 1000 bp DNA marker; Lane 1–23: tested field samples.

Among 47 clinical samples, mixed infections involving two or more bacterial species were identified in 35/47 (74.5%) samples, whereas single‐pathogen infections were observed in 12/47 (25.5%) samples. To be specific, *E. coli* was detected in 38/47 (80.8%) samples, *K. pneumoniae* in 31/47 (65.9%), *P. aeruginosa* in 19/47 (40.4%), and *P. multocida* in 19/47 (40.4%). The multiplex PCR results were in complete agreement with those obtained by 16S rRNA sequencing shown in Table 2. Random selected detection and identification results are shown in Figure 5.

**TABLE 2:** Comparison of multiplex PCR and 16S rRNA sequencing for the detection of four bacterial pathogens in 47 clinical samples.

Pathogens	Proportion of positive samples		Concordance rate
	Multiplex PCR	16S rRNA sequencing	
*E. coli*	38/47 (80.8%)	38/47 (80.8%)	100%
*K. pneumoniae*	31/47 (65.9%)	31/47 (65.9%)	100%
*P. aeruginosa*	19/47 (40.4%)	19/47 (40.4%)	100%
*P. multocida*	19/47 (40.4%)	19/47 (40.4%)	100%

### 3.7. Results of Histopathological Analysis for Field Samples

**FIGURE 6:**
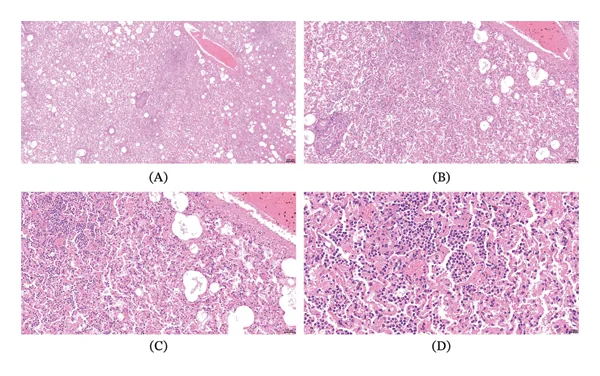
Histopathological examination of lung tissue from a 1.5‐year‐old female forest musk deer by hematoxylin and eosin (H&E) staining. (A) Lung sections exhibited marked consolidation, characterized by alveolar spaces filled with exudates and inflammatory cells, resulting in partial or complete loss of the normal air‐containing alveolar architecture, a typical feature of severe pulmonary inflammation or pneumonia. (B) Pronounced vascular congestion was observed, as indicated by blood vessels engorged with erythrocytes, reflecting local circulatory stasis and an active inflammatory response commonly seen in acute inflammation or infection. (C) Evident alveolar septal thickening was present, likely associated with inflammatory cell infiltration, interstitial edema, or fibrin deposition, indicating impaired gas exchange. (D) Extensive infiltration of polymorphonuclear neutrophils was observed, representing a hallmark pathological feature of acute bacterial infection and suppurative inflammation.

Lung sections revealed profound parenchymal consolidation and extensive architectural disruption of the pulmonary tissue as shown in Figure 6. At low magnification, the alveolar lumina were predominantly obliterated by a dense hemorrhagic‐purulent exudate, indicative of severe exudative bronchopneumonia. High‐power microscopic examination confirmed a massive leukocytic infiltration, primarily composed of polymorphonuclear neutrophils and activated macrophages, interspersed within a proteinaceous fibrinous meshwork. Furthermore, marked capillary hyperemia and widespread intra‐alveolar hemorrhage were evident, accompanied by significant interstitial edema and alveolar septal thickening. These acute suppurative‐hemorrhagic lesions are pathognomonic for severe infection in the respiratory tract, providing robust morphological evidence that correlates with the high detection frequencies of the targeted pathogens via the established multiplex PCR assay.

## 4. Discussion

Since ancient times, musk has been widely used in traditional Chinese medicine, perfumery, and religious rituals because of its unique fragrance and remarkable medicinal properties, resulting in persistently high demand. At present, captive breeding of forest musk deer represents the only legal means of obtaining musk. With the expansion of captive breeding, the impact of pathogenic microorganisms on forest musk deer farming has received increasing attention. Musk has long been valued in traditional Chinese medicine, perfumery, and religious rituals, and breeding of forest musk deer is currently the only legal source of musk. As forest musk deer breeding has expanded, increasing attention has been paid to diseases affecting deer. Among these, respiratory diseases are a major health concern because of their high mortality. An analysis of 339 mortality cases by Wang and Meng showed that respiratory diseases accounted for the highest mortality and affected all age groups [31], highlighting the importance of effective disease prevention and control in captive populations.

Multiplex PCR technology enables the simultaneous identification of multiple pathogenic microorganisms in a single reaction, allowing rapid and accurate pathogen detection. This approach greatly improves the timeliness of etiological diagnosis and facilitates the early implementation of targeted prevention and control measures, thereby providing important support for epidemiological surveillance, formulation of clinical treatment strategies, and overall improvement in disease control efficiency [32, 33]. The target genes selected in this study were chosen based on three criteria: (i) high sequence conservation within each bacterial species, (ii) sufficient divergence from homologous genes in closely related bacteria to ensure specificity, and (iii) previous validation in molecular diagnostic assays. First, *gyrB* and *phoE* are housekeeping genes that exhibit high intraspecies conservation while maintaining sufficient interspecies sequence divergence, and both are included among the seven loci used for multilocus sequence typing (MLST), highlighting their sequence stability and suitability for species‐specific molecular detection [34, 35]. Second, though the 16S rRNA gene has been widely used for bacterial identification, its limited sequence variability often restricts discrimination among closely related bacterial species. Third, studies have shown that the *LasI* gene sequence is highly conserved among different serotypes of *P. aeruginosa* [36], making it a suitable target for species identification. The *Kmt1* gene has been widely used as a specific target for the identification of *P. multocida* in PCR‐based assays [37–39]. Collectively, these target genes possess favorable characteristics for multiplex PCR, including high conservation, species specificity, stable evolutionary characteristics, and extensive validation in previous diagnostic studies, thereby supporting their suitability for simultaneous detection of the four bacterial pathogens.

Precise optimization of annealing temperature and appropriate adjustment of primer concentration are critical for ensuring the specificity, sensitivity, and overall accuracy of PCR amplification. When designing specific primers, it is essential to ensure that the melting temperatures (Tm) of the four primer pairs are similar and that their GC contents fall within a comparable range, thereby reducing the formation of primer dimers and hairpin structures [40].

The annealing temperature for conventional PCR generally ranges from 55°C to 72°C. Consistent with this, amplification efficiency remained stable across the tested temperature range (55°C–65°C), as indicated by agarose gel electrophoresis. Therefore, 60°C was selected as the optimal annealing temperature based on overall amplification performance and practical considerations.

In experiments involving multiple primer pairs, researchers often dilute or group primers individually. However, in this study, each target template corresponded to only one primer pair, resulting in a relatively simple experimental system. Therefore, the four primer pairs were mixed prior to dilution, which effectively reduced operational complexity. The primer stock concentration was finally determined to be 0.625 μM. Under these conditions, optimal amplification efficiency and high cost‐effectiveness were achieved.

Sensitivity is a critical parameter in multiplex PCR assays, as it directly affects the reliability, accuracy, and applicability of detection results. Bottero established a multiplex PCR method for detecting *E. coli* in dairy products and reported varying sensitivities among different target genes: the detection limits for *hlyA*, *st*, *eaeA*, and *vt1* were 5 × 10^4^ CFU/mL, whereas those for *lt* and *vt2* were 1 × 10^6^ CFU/mL [41]. Khazani developed a duplex PCR assay for the detection of *K. pneumoniae* and *Haemophilus influenzae*, targeting the *php* gene of *K. pneumoniae*, with a detection limit of 1 × 10^4^ CFU/mL [42]. Wang designed novel target genes for *P. aeruginosa* and established a singleplex PCR assay using four primer pairs (PA1–PA4), with detection limits of 4.1 × 10^5^ CFU/mL, 9.7 × 10^3^ CFU/mL, 4.3 × 10^4^ CFU/mL, and 4.3 × 10^4^ CFU/mL, respectively [43]. In another study, Wang established a multiplex PCR method for avian bacterial diseases and designed specific primers targeting the *Kmt1* gene of *P. multocida*, achieving a detection limit of 2.8 × 10^3^ CFU/mL [44]. In the present study, the detection limits, in a 25 μL PCR reaction volume, were 9.2 × 10^4^ CFU/mL for *E. coli*, 1.3 × 10^5^ CFU/mL for *K. pneumoniae*, 1.5 × 10^5^ CFU/mL for *P. aeruginosa*, and 3.7 × 10^4^ CFU/mL for *P. multocida*. The detection limits obtained were not only consistent with those reported in previous studies but also comparable to those. It directly confirms the reliability and robustness of the present method.

Clinical samples often harbor complex pathogen profiles, and the detection efficiency of multiplex PCR far exceeds that of the “gold standard” (bacterial isolation and identification methods). After reviewing existing multiplex PCR assays, we found that no multiplex PCR method targeting pathogens associated with forest musk deer pneumonia has been reported to date. The multiplex PCR assay established in this study enables rapid detection of four major bacterial pathogens. To comprehensively validate the established multiplex PCR assay, 47 clinical tissue samples were subjected to multiplex PCR detection, bacterial isolation followed by 16S rRNA sequencing, and histopathological examination. The detection rates of *E. coli* (80.8%), *K. pneumoniae* (65.9%), *P. aeruginosa* (40.4%), and *P. multocida* (40.4%) obtained by multiplex PCR were completely consistent with those identified by bacterial isolation and 16S rRNA sequencing, demonstrating the high accuracy and reliability of the assay. In addition, histopathological analysis of the lung tissues revealed acute suppurative and hemorrhagic lesions typical of severe bacterial respiratory septicemia. The concordance among molecular detection, microbiological identification, and pathological findings provides robust evidence supporting the reliability, feasibility, and clinical utility of the established multiplex PCR assay for the diagnosis of bacterial pneumonia in forest musk deer.

Compared with traditional bacterial isolation methods and single‐target PCR assays, multiplex PCR allows simultaneous amplification of multiple pathogens, greatly improving detection efficiency and better meeting the requirements of clinical disease prevention and control. Furthermore, the flexibility and scalability of multiplex PCR enable adaptation to changes in pathogen composition, further enhancing its potential application in epidemiological surveillance. By optimizing primer design and reaction conditions, multiplex PCR not only improves sensitivity and specificity but also provides a feasible solution for the simultaneous detection of multiple targets, offering robust data support for disease prevention and control. However, bacterial infections still require appropriate antibiotic therapy.

## Funding

This study was supported by the Natural Science Foundation of Jilin Province (YDZJ202501ZYTS554) and the Performance Task Statement for the Leap Development Phase of the Science and Technology Innovation Project of the Chinese Academy of Agricultural Sciences (CAAS‐ASTIP‐2021‐ISAPS).

## Conflicts of Interest

The authors declare no conflicts of interest.

## Data Availability

Data sharing is not applicable to this article as no datasets were generated or analyzed during the current study.
